# Supplementary material for: Comparing the fit of N95, KN95, surgical, and cloth face masks and assessing the accuracy of fit checking
Source: PLoS One. 2021 Jan 22;16(1):e0245688. doi: 10.1371/journal.pone.0245688 (PMC7822328; doi:10.1371/journal.pone.0245688)
Supplement: Attachment — Submitted filename: Reviewer 3 Response.pdf [file pone.0245688.s002.pdf]

Dear Reviewer 3,

Firstly, thank you for taking the time to review and provide feedback on our article. We have made multiple changes as suggested by you and the other reviewers. For your convenience, we have broken down our response according to each of your comments.

In addition to minor changes, we have made several major changes including:

- More detailed literature analysis
- Clarifying the experimental design, including two new figures
- Clarifying importance of results, including findings not previously discussed such as the impact of minor facial feature differences on fit

Reviewer Comment	Response or Change
Have the authors made all data underlying their findings in their manuscript fully available? - No	We apologize if this was not clear in the submission file. All data we collected is available in an open data repository. The DOI for this data set is: https://doi.org/10.17863/CAM.56361. This information is listed at the very end of the article under the "Supporting Information" heading.
The conclusion that, "Some respirators don't fit" is not very helpful.	We have made some changes to the conclusion to clarify the main contributions of the paper.
What is the reproducibility within one subject? That is, have them put it on and off.	Typical fit factor accuracy given by the Portacount 8038 is +/- 10% of reading.<br><br>It is assumed that test results are reproducible – that is the reasoning behind a person only having to pass a fit test once before an N95 is deemed safe for use in clinical practice. It is recognized that tests are not reproducible when there are changes to face shape or features. Given that the tests in this study were conducted over a very short time frame, any facial changes are extremely unlikely.<br><br>The reproducibility of quantitative fit test results for poorly fitting masks (such as surgical masks) is an area of research being explored by this research group as part of a separate study. Our results indicate that tests are generally accurate within a range of less

Reviewer Comment	Response or Change
	than 10% when testing N95 masks and +/- 10% for loose-fitting masks. In a few fabric masks, the variation could extend to +/- 15%. We observed that these masks tended to slip and move about on the face and/or where masks that required to be tied on with ribbons (which tended to come loose over time).
Different races and ethnicities have different face shapes. Could this be impacting the fit?	Anything that effects face shape and features will affect mask fit. Race, gender, ethnicities – all of these impact face shapes. Our sampling method also allowed us to compare differences between genetically related individuals. We have added how a new section discussing how even small differences (such as the height of a nose bridge, the fullness of a cheek or chin) can have a drastic impact on fit. Two participants we tested were related - participant F-68 was the mother of F-28. They had very similar bone structure, but F-28, being younger, had more subcutaneous fat under the chin. This small difference created a significant difference in fit factor for N95 masks. We have now included a discussion of the impact of these minor differences in the paper.
How does fit change over time? Or with decontamination?	This is an interesting question but outside the scope of our study. Most N95 decontamination methods are known to impair the filtration ability of a mask, thus reducing its fit factor. This is why we did not try to decontaminate masks, but used instead a mask for each participant. We are investigating fit changes over time for a future study, and early results show that fit is most heavily impacted by activity, with more vigorous activities occasionally breaking the mask's seal.
Qualitative fit testing can use odorants.	You are very right - and I just found several locations were we said we were conducting qualitative rather than quantitative fit testing. I am surprised and ashamed our group did not catch that during our many rounds of editing. We performed quantitative fit testing only.

Reviewer Comment	Response or Change
The figures are very blurry. Not publishable in current state.	I believe this is due to the way PlosONE compresses images for the PDF preview. The following is taken form the PlosONE website:<br><br>"The compiled submission PDF includes low-resolution preview images of the figures after the reference list. The function of these previews is to allow you to download the entire submission as quickly as possible. Click the link at the top of each preview page to download a high-resolution version of each figure. Links to download Supporting Information files are also available after the reference list."
