# Supplementary material for: Comparing the fit of N95, KN95, surgical, and cloth face masks and assessing the accuracy of fit checking
Source: PLoS One. 2021 Jan 22;16(1):e0245688. doi: 10.1371/journal.pone.0245688 (PMC7822328; doi:10.1371/journal.pone.0245688)
Supplement: Attachment — Submitted filename: paper review_PLOS ONE_1121.pdf [file pone.0245688.s003.pdf]

This manuscript introduced the fitting tests of N95, KN95, Surgical and cloth face masks, and evaluated the accuracy of fit checking, emphasizing the importance of fitting of face coverings. The test protocol includes a fit check and quantitative fit testing. This study stresses the significance of fit, which is critical to the current COVID-19 pandemic. The technical quality of the manuscript has improved after the previous revision, and the authors have sufficiently addressed the comments from the previous reviewers. The reviewer recommends the publication of this manuscript after minor revisions.

General comments:

1. It is recommended to replace “N95 and KN95 masks” with “N95 and KN95 respirators” throughout the paper because masks are not designed for complete sealing.

Specific comments:

1. Page 4: the full name of CDC is the Centers for Disease Control and Prevention.
2. Page 12 and 17: it will be very helpful to illustrate the impact of minor facial differences on the fit of the respirators. It will be good to compare more fit factor scores on page 12, instead of qualitative comparison only. Also, please quantify the subcutaneous fat under the chin if possible. The authors may consider adding “bone structure and nose length and width” to page 17 as your examined factors.
