# Supplementary material for: Comparing the fit of N95, KN95, surgical, and cloth face masks and assessing the accuracy of fit checking
Source: PLoS One. 2021 Jan 22;16(1):e0245688. doi: 10.1371/journal.pone.0245688 (PMC7822328; doi:10.1371/journal.pone.0245688)
Supplement: Attachment — Submitted filename: reviewer responses.docx [file pone.0245688.s004.docx]

We want to thank the reviewers again for their continued time and effort in helping us improve this manuscript. We have made all the requested changes. These include:

- Replace the word “mask” with “respirator” when discussing N95s or KN95s. We have continued to use the term “mask” to refer to fabric and surgical coverings.
- Added “and Prevention” to the CDC’s full title
- Added details and measurements to better illustrate how very minor physical differences impact fit
- Corrected two figure references
